# Adrenalin in General Surgery

**Published:** 1904-01

**Authors:** 


### Adrenalin in General Surgery.

HARRY GIDNEY, F. R. C. S. (Edin), (*Indiana Med. Gazette,* October, 1903,) finds the clinical usefulness of adrenalin is very great and extensive, and owing to its power of rapidly and effectively producing vasomotor constriction, it is adapted to the treatment of almost all inflammatory conditions. The drug is also of extreme value in arresting hemorrhage during surgical operations. It is indicated whenever and wherever any local hyperemia exists, more especially in inflammations of mucous surfaces such as those of the eye, throat, larynx, pharynx, urethra, bladder, nose, rectum, vagina, uterus, stomach, and the like. It is used not only to stay hemorrhage when it exists, but also as a preventive or controlling remedy, given either internally or externally prior to an operation, so as to lessen the amount of bleeding during operation procedures. It is nonirritant to mucous membranes unless used too frequently and in excess.

On reading the literature on the subject, says the writer, I find that adrenalin is admitted to be the most powerful and rapid cardiac stimulant and tonic we have, being chiefly used in cardiac affections, hematemesis, hemoptysis, hemophilia, hematuria, menorrhagia, post-partum hemorrhage, purpura, scurvy, and in similar conditions. It is said to be the most rapid restorative in chloroform and other forms of anesthetic syncope, and in such cases it is advisable to administer it intravenously. The author reports the results of several operations, major and minor, in which adrenalin was employed. The first case was one of fracture of the vertex of the skull. As one of the larger branches of the middle meningeal artery had been torn there was profuse dural hemorrhage and capillary oozing which were controlled by the use of the 1-1000 solution. In the second case, one of hemorrhoids, profuse bleeding was checked by the rectal insertion of a plug of cotton wool, soaked with adrenalin chloride solution.

The third case was one of skin grafting in which the author tried pressure to stop the capillary bleeding. As the procedure was somewhat tedious he applied adrenalin chloride solution with almost immediate cessation of all oozing, and what is usually a lengthy and sanguinary operation was converted into a short comparatively bloodless one. The fourth case, one of hemorrhage after the extraction of teeth, and the fifth, which appears to embrace the author's experience in a number of cases of epistaxis, afforded additional opportunity to test the hemostatic effect of adrenalin. In the sixth case a post-partum hemorrhage was checked by swabbing the uterine cavity with adrenalin solution, while the same happy result was obtained in a case of secondary hemorrhage following an operation for the relief of a mammary abscess.

Gidney has found that the instillation of a 1-5000 to 1-2000 solution of this drug reduces the inflammation and considerably cuts short the process of conjunctivitis. He usually applies it (diluted) over the inflamed parts by means of a soft camel's hair brush. He always uses the preparation containing chloretone, which has a decided local anesthetic action, relieving much of the photophobia and pain. He is fully convinced of the power of adrenalin to arrest or lessen the bleeding that arises from the cut ends of the iris after iridectomy. He speaks highly of its efficiency in chemosis, cataract operations, evisceration of the eyeball, operations for ectropion, symblepharon and trachomatous pannus.

The author concludes that in all cases of minor surgery in which it is desired to arrest bleeding from any cut or exposed surface we have in adrenalin a most useful, powerful and rapid drug,—one that is not poisonous, nonirritant and not accumulative in action, especially in operations upon the conjunctiva and eyelids.

---

Note.—Credit for this abstract should read Indian, instead of Indiana Med. Gazette.

